# The Use of the Surveyor in Removable Partial Denture Prosthetics: A Survey Among Dental Technicians in Casablanca

**DOI:** 10.7759/cureus.113494

**Published:** 2026-07-28

**Authors:** Aicha Oubbaih, Bahia Bouanane, Bouchra Badre, Basma Stitou, Yasmina Cheikh, Khadija Kaoun, Samira Bellemkhannate

**Affiliations:** 1 Department of Removable Prosthodontics, Faculty of Dental Medicine, Hassan II University of Casablanca, Casablanca, MAR; 2 Laboratory of Community Health, Epidemiology and Biostatistics, Hassan II University of Casablanca, Casablanca, MAR

**Keywords:** casablanca, dental surveyor, dental technicians, laboratory practices, removable partial dentures, survey

## Abstract

Introduction

Preserving oral structures in removable partial denture (RPD) therapy requires meticulous surveying analysis to ensure long-term clinical success. This study aimed to examine how dental surveyors are utilized across dental laboratories in Casablanca.

Materials and methods

We conducted a descriptive cross-sectional survey targeting 105 private-practice dental technicians in Casablanca, Morocco, over a three-month period (October 2023 to January 2024). Data regarding professional backgrounds, framework design protocols, and surveying practices were collected using a structured 20-item questionnaire administered by telephone and in physical and digital formats. Data were analyzed using descriptive statistics (frequencies and percentages) in SPSS Statistics for Windows, version 16.0 (released 2007; SPSS Inc., Chicago, IL, USA).

Results

Most respondents were experienced men (71.4%; 81% with >10 years in practice), yet only 43.8% actively participated in continuing education. Although 71.2% of the laboratories owned a surveyor, its routine use remained low at 30.5%, with nearly half (47.6%) of the respondents considering the tool optional. Furthermore, a major gap in clinician-laboratory communication was identified: 81.9% of technicians independently designed the RPD frameworks, even though only 8.6% of the prescribing clinicians had a surveyor in their private offices.

Conclusions

The use of the dental surveyor in Casablanca is characterized by a significant gap between equipment ownership and clinical practice. Insufficient continuing education and a lack of communication between practitioners and laboratories highlight the urgent need for better coordination to ensure the quality of prosthetic rehabilitation.

## Introduction

Removable partial denture (RPD) therapy must balance the restoration of masticatory function and esthetics with the preservation of the remaining oral tissues. Although restoring edentulous spaces may appear straightforward, achieving long-term therapeutic success depends on a carefully engineered framework design based on a meticulous preprosthetic assessment [1].

The path of insertion and removal must be precisely determined to prevent the generation of harmful lateral or torquing forces on the abutment teeth during placement and removal of the prosthesis. Achieving this requires the systematic use of a dental surveyor at two critical stages: first, chairside, to guide the intraoral preparation of guiding planes and rest seats on the abutment teeth; and second, in the laboratory, during the design and casting of the metal framework, to accurately position the retentive clasps, reciprocal arms, and minor connectors relative to the previously established path of insertion [2].

The surveyor identifies the relative parallelism of supporting surfaces and detects undercut areas. Failure to use this instrument compromises the accuracy of the intended path of insertion, which may result in improper seating of the prosthesis and often necessitates additional, uncontrolled reduction of the RPD guiding planes intraorally to achieve a passive fit. It also poses significant biological risks, particularly the uneven distribution of masticatory forces, which can lead to osteolysis, soft tissue lesions, or abutment tooth mobility [3].

Although conventional, nondigital techniques remain widely used in daily practice, the systematic use of the dental surveyor is often neglected by laboratory technicians. This underutilization is well documented in the international literature. Surveys conducted in the United Kingdom and Sudan have highlighted significant deficiencies in communication and low adherence to strict surveying protocols during RPD fabrication [4,5]. In Morocco, however, there are no published data regarding these laboratory practices. Assessing these practices is crucial to understand the extent of this underutilization, protect the clinical success of RPDs, and guide future professional training. Therefore, the objective of this study was to investigate adherence to surveying protocols during RPD design in dental laboratories in Casablanca.

## Materials and methods

Study design and setting

A cross-sectional, descriptive epidemiological study was conducted among dental technicians operating in the private sector in Casablanca, Morocco. The survey was carried out over a three-month period, from October 23, 2023, to January 28, 2024.

Study population

The study population was selected through random sampling from a publicly available commercial business directory listing private-sector dental technicians in Casablanca, given the absence of a centralized, officially certified registry of practicing dental technicians in the region. Because the exact total number of practicing technicians in Casablanca could not be determined, a formal a priori sample size calculation was not feasible. Instead, all eligible technicians listed in the directory were considered, and 170 were randomly selected and contacted for participation. Of these, a final sample of 105 dental technicians successfully completed the survey and were included in the final analysis, representing a response rate of 61.8%.

Inclusion and exclusion criteria

To be included in the study, participants had to be private-sector technicians whose routine laboratory practice actively included the fabrication of RPDs. Exclusion criteria included permanently closed laboratories that could not be contacted or surveyed and technicians exclusively employed in hospital or university settings, as their institutional practice conditions, characterized by institutional supervision, standardized protocols, and different equipment access, differ substantially from those of independent private laboratories. These differences could confound the assessment of routine surveying habits in the private sector.

Data collection

Data collection was performed using a structured 20-item questionnaire specifically developed for this study (Appendix A). To ensure content validity, the instrument was designed based on a comprehensive review of the literature and validated by a panel of academic experts in prosthodontics at our institution. Prior to deployment, a pilot study was conducted with a small group of dental technicians (n = 10), who were excluded from the final analysis, to assess the clarity and comprehension of the questions. Minor adjustments were made accordingly.

The instrument was divided into two primary sections. The first section gathered data regarding the professional profile of the participants, including demographics, formal education, clinical experience, and participation in continuing education. The second section focused on surveyor utilization, evaluating the proportion of RPDs in overall practice, specific design techniques, the frequency and type of dental surveyor used, encountered technical difficulties, prosthetic remake rates, and perceived training needs. To optimize the response rate and ensure comprehensive data acquisition, the questionnaire was administered using multiple modalities, including telephone interviews, physical hard copies, and digital formats. Missing data were managed by applying strict completeness criteria; only fully completed questionnaires (n = 105) were included in the final analysis, whereas incomplete forms were excluded.

Statistical analysis

Statistical analysis was performed at the Epidemiology Laboratory of the Faculty of Dentistry of Casablanca using SPSS Statistics for Windows, version 16.0 (released 2007; SPSS Inc., Chicago, IL, USA). Descriptive statistics were applied to summarize all evaluated variables. Qualitative variables were expressed as absolute frequencies and percentages, whereas quantitative variables were presented as means with corresponding standard deviations.

## Results

The results of this study provide an overview of dental surveyor utilization among 105 surveyed private-practice dental technicians in Casablanca.

Demographic and professional profile

The study population was predominantly male (71.4%), with a significant proportion aged between 30 and 40 years (44.8%). The workforce was highly experienced, with 81% of respondents having more than 10 years of professional practice. Although 75.2% graduated from private institutions, only 43.8% actively participated in continuing education programs (Table 1).

**Table 1:** Demographic and professional profile of the study population. FMDC, Faculté de Médecine Dentaire de Casablanca; FMDR, Faculté de Médecine Dentaire de Rabat

Variable	Count (n)	Percentage (%)
Gender		
Male	75	71.4
Female	30	28.6
Age		
20-30 years	17	16.2
30-40 years	47	44.8
40-50 years	23	21.9
>50 years	18	17.1
Training institution		
FMDC	12	11.4
FMDR	0	0
Private institution	79	75.2
Other	14	13.3
Year of graduation		
1983-1992	11	10.5
1992-2001	22	21
2001-2010	37	35.2
2010-2019	28	26.6
No response	7	6.7
Professional experience		
<10 years	20	19
10-20 years	44	41.9
>20 years	41	39
Continuing education attendance		
Yes	46	43.8
No	59	56.2
Completed training programs		
Implant-supported prosthetics	7	6.7
Ceramics	7	6.7
3D digital technology	26	24.8
Veneers	5	4.8
No response	60	57.1
Total	105	100

Surveyor utilization in laboratories

Although a clear majority of laboratories owned a dental surveyor (71.2%), its routine use remained very low, with only 30.5% of technicians using it systematically for all cases. In contrast, 39% reported never using it. Among laboratories equipped with a surveyor, conventional analog parallelometers largely dominated the workflow (81%) compared with digital systems (19%) (Table 2).

**Table 2:** Use of the surveyor within dental prosthesis laboratories. Percentages may not sum to 100% due to rounding. CAD/CAM, computer-aided design/computer-aided manufacturing; RPD, removable partial denture

Variable	Count (n)	Percentage (%)
Equipment with a surveyor		
Yes	75	71.2
No	30	28.8
Type of surveyor		
Digital	20	19
Conventional	85	81
Surveyor brand		
BEGO	18	17.1
TECH-DENT	35	33.3
Schick Dental	3	2.9
CAD-CAM	3	2.9
Digital system	7	6.4
BIO ART	2	2
Frequency of use		
Always	32	30.5
Often	23	21.9
Rarely	9	8.6
Never	41	39
Surveyor usage		
All RPD cases	46	43.8
Complex cases only	19	18.1
No response	40	38.1
Total	105	100

Difficulties and perceived impact

Time constraints were identified as the primary operational drawback by 47.6% of the cohort, followed by the perceived complexity of the tool (12.4%). Furthermore, significant professional inertia was observed, as 59% of technicians believed that a surveyor did not provide a valuable contribution to their work, and 59% stated that prosthetic remakes due to its nonuse occurred only rarely (Table 3).

**Table 3:** Difficulties related to the use of the surveyor. Percentages may not sum to 100% due to rounding.

Variable	Count (n)	Percentage (%)
Drawbacks of using the surveyor		
Time	50	47.6
Complexity	13	12.4
Other	38	36.9
Percentage of dentures remade due to nonuse of the surveyor		
Often	1	1
Rarely	62	59
Never	42	40
Dental technicians’ awareness of the surveyor’s contribution		
Yes	43	41
No	60	59
Total	105	100

Clinical-technical interface

A significant communication gap exists between clinicians and dental laboratories. Only 8.6% of prescribing dentists own a surveyor in their private offices, and 66.7% do not mandate its use. Consequently, responsibility for designing the RPD framework outline is fully delegated to the dental technician in 81.9% of cases (Table 4).

**Table 4:** Use of the surveyor by dentists. Percentages may not sum to 100% due to rounding.

Variable	Count (n)	Percentage (%)
Dentists’ possession of a surveyor		
Yes	9	8.6
No	96	91.4
Requirement for surveyor use by dentists		
Yes	35	33.3
No	70	66.7
Designing the frame’s outline		
By the dentist	4	3.8
By the dental technician	86	81.9
No difference	15	14.3
Total	105	100

## Discussion

The primary objective of this study was to evaluate adherence to dental surveying protocols and investigate how these critical instruments are utilized across private dental laboratories in Casablanca. RPD therapy requires a meticulous balance between restoring function and preserving remaining oral structures, a success that hinges on objective surveyor-guided planning. Our findings reveal a significant paradox within the local dental ecosystem: a pronounced discrepancy between high equipment ownership and deficient routine clinical implementation, largely driven by educational gaps and a fragmented clinical-technical interface.

Regarding demographic characteristics, the striking male predominance observed among private-sector technicians (71.4%) reflects a structural trend consistent with earlier data collected across Rabat and Casablanca, where studies reported 74% and 62.8% male predominance, respectively [6,7], and closely mirrors the 78% male predominance reported among registered technicians in Dakar [8]. Academically, although the vast majority of the cohort was trained within private Moroccan institutions, a concerning segment operates without formal qualifications, relying entirely on empirical, on-the-job apprenticeship. Although this workforce is highly experienced, with a substantial majority having more than a decade of active practice, this professional longevity does not automatically guarantee updated competencies. Professional field experience is a critical determinant of prosthetic manufacturing quality, a trend closely echoing observations documented among a similarly experienced technician cohort in Dakar [8]. However, without standardized continuing education, local clinical practices risk stagnating with legacy methods rather than evolving according to contemporary guidelines.

This adherence to traditional paradigms is further illustrated by the fact that conventional surveyors remain deeply entrenched in local workflows, leaving digital surveying systems vastly marginalized. This heavy reliance on traditional hardware contrasts with the rapid digital transition occurring across the international dental sector. In France, for instance, the adoption of computer-aided design/computer-aided manufacturing systems in removable prosthodontics has progressed considerably in recent years, offering new perspectives for both complete and partial dentures without fully replacing traditional analog methods [9]. Modern digital surveying software replaces physical gauges with interactive, color-coded undercut mappings, enabling highly dynamic and intuitive assessment of insertion axes [10,11]. Nevertheless, analog alternatives demonstrate remarkable local resilience, a phenomenon also explicitly noted in Reims, where conventional surveyors maintain a firm foothold in daily routines [12]. This enduring popularity may be explained by negligible overhead costs and time-tested mechanical reliability. However, treating the surveyor as a discretionary accessory compromises casting precision and clinical fit, a pattern also documented in French cohorts, where comparative studies on path-of-insertion determination methods have shown that this step is frequently delegated to technicians due to its perceived complexity and that insufficient clinical data collection is a key obstacle to reproducible, successful RPD treatment outcomes [13,14].

Beyond technological preferences, severe time constraints and perceived operational complexity emerged as the primary roadblocks to systematic integration. This professional inertia reflects an underestimation of the biological and mechanical trauma that an unguided framework can inflict on supporting oral tissues. These specific bottlenecks align with long-standing trends identified in French literature, which consistently highlight deficient initial training, operational time constraints, and fragmented communication as core limiting factors [15,16]. Without explicit, clinically derived path-of-insertion guidance provided chairside by the prescribing dentist, laboratory surveying becomes an unguided process. Furthermore, the low rate of immediate laboratory remakes reported by technicians introduces a potential perceptual bias. The biological consequences of an unguided design may accumulate silently over months or years, manifesting as abutment tooth wear, bone loss, and chronic soft-tissue trauma under cyclic masticatory loading. Consequently, low laboratory return metrics may mask the true clinical impact of deficient preprosthetic planning, a concern supported by recent systematic reviews showing high rates of long-term patient dissatisfaction and prosthetic abandonment [17].

This professional gap is deeply rooted in the clinical-technical interface, characterized by limited clinician involvement in framework design and the transfer of responsibility for drawing RPD outlines to dental technicians. This systemic delegation is directly related to a substantial hardware deficit in clinical settings, where only a small proportion of prescribing dentists (8.6%) own a surveyor. This pattern represents a documented global phenomenon: in a French survey, only 12% of practitioners reported using a surveyor and personally tracing their framework design, a proportion comparable to the 18.1% of Moroccan clinicians in our sample who retained some involvement in RPD design [15]. Similarly low clinician involvement in RPD design and surveying has also been reported in Ireland and the United Kingdom [4] and in Sudan, where dentists provided no design-related instructions to laboratories in 21.7% of cases, despite 84.2% of technicians believing that design responsibility should rest with the dentist [5]. Determining the path of insertion is fundamentally a diagnostic clinical decision, not a mechanical afterthought, as it directly dictates the position of guiding planes, retentive components, and the long-term survival of the remaining dentition [18,19]. Delegating this step without adequate clinical guidance transfers medical responsibility to a technical partner who lacks direct access to the patient’s periodontal health, occlusal dynamics, and soft-tissue architecture.

Finally, this investigation has several methodological limitations. First, calculating an optimal sample size was hindered by the absence of a centralized registry of practicing dental technicians in Casablanca, making systematic identification of the target population challenging. Furthermore, data collection frequently conflicted with the demanding schedules of participants. In addition, concerns regarding professional and legal confidentiality made some technicians hesitant to participate, potentially introducing nonresponse bias and prolonging the data collection period.

## Conclusions

This study evaluated the utilization of dental surveyors among private dental technicians in Casablanca, revealing a significant gap between high equipment availability and deficient routine implementation in laboratory workflows. Although conventional surveyors remain physically available in most laboratories, substantial time constraints, perceived operational complexity, and insufficient clinical guidance lead technicians to fabricate RPD frameworks without objective planning. Addressing this critical gap requires standardized continuing education tailored specifically for dental technicians, alongside a restructured clinical-technical interface that promotes shared responsibility and clear communication guidelines. Ultimately, resolving these operational barriers is essential to transition local laboratory practices from empirical approaches to contemporary, quality-assured prosthetic standards.

## Appendices

Appendix A: Study questionnaire

Part 1: Identification

1. Gender

☐ Female

☐ Male

2. Age

☐ 20-30 years

☐ 30-40 years

☐ 40-50 years

☐ Over 50 years

3. Training institution

☐ Faculty of Dental Medicine - Casablanca

☐ Faculty of Dental Medicine - Rabat

☐ Private institution

☐ Other (please specify): __________________________________

4. Year of graduation __________________________________

5. Years of professional experience

☐ Less than 10 years

☐ Between 10 and 20 years

☐ More than 20 years

6. Do you participate in continuing education?

☐ Yes

☐ No

If yes, please specify the training(s) attended: __________________________________

Part 2: Use of the Surveyor in the Laboratory

7. What proportion of your laboratory work involves removable partial dentures (RPDs)?

☐ 0-25%

☐ 25-50%

☐ 50-75%

☐ 75-100%

8. When designing an RPD, which technique do you use?

☐ Conventional technique

☐ Digital technique

☐ Both

9. Do you have a surveyor?

☐ Yes

☐ No

If no, why not? __________________________________

10. What type of surveyor do you use?

☐ Conventional

☐ Digital

11. What brand of surveyor do you use? __________________________________

12. If you use it, it is for:

☐ All RPD cases

☐ Complex cases only

☐ Other (please specify): __________________________________

13. When fabricating an RPD, how frequently do you use a surveyor?

☐ Always          ☐ Often

☐ Rarely            ☐ Never

14. Does your referring dentist have a surveyor?

☐ Yes

☐ No

15. Does your referring dentist require surveyor use?

☐ Yes

☐ No

If no, why not? __________________________________

16. In your opinion, who should design the framework outline?

☐ The dentist

☐ The dental technician

☐ It makes no difference

17. Do you think the surveyor has limitations?

☐ Time-consuming          ☐ Difficult to use

☐ Cost                              ☐ Other

18. Do you receive feedback from the referring dentist regarding prosthesis insertion problems?

☐ Always          ☐ Rarely

☐ Often             ☐ Never

19. In your opinion, could these problems be prevented by using a surveyor?

☐ Yes - why? __________________________________

☐ No - why not? __________________________________

20. Would you like to participate in continuing education on the use of the surveyor in prosthetics?

☐ Yes

☐ No
